# Complete Models of p53 Better Inform the Impact of Hotspot Mutations

**DOI:** 10.3390/ijms232315267

**Published:** 2022-12-03

**Authors:** Maria J. Solares, Deborah F. Kelly

**Affiliations:** 1 Molecular, Cellular, and Integrative Biosciences Graduate Program, Huck Institutes of the Life Sciences, Pennsylvania State University, University Park, PA 16802, USA; 2 Department of Biomedical Engineering, Pennsylvania State University, University Park, PA 16802, USA; 3 Center for Structural Oncology, Pennsylvania State University, University Park, PA 16802, USA

**Keywords:** p53, cancer, molecular modeling, tumor suppressor, apoptosis, DNA repair

## Abstract

Mutations in tumor suppressor genes often lead to cancerous phenotypes. Current treatments leverage signaling pathways that are often compromised by disease-derived deficiencies in tumor suppressors. P53 falls into this category as genetic mutations lead to physical changes in the protein that impact multiple cellular pathways. Here, we show the first complete structural models of mutated p53 to reveal how hotspot mutations physically deviate from the wild-type protein. We employed a recently determined structure for the p53 monomer to map seven frequent clinical mutations using computational modeling approaches. Results showed that missense mutations often changed the conformational structure of p53 in the DNA-binding site along with its electrostatic surface charges. We posit these changes may amplify the toxic effects of these hotspot mutations by destabilizing an important zinc ion coordination region in p53 to impede proper DNA interactions. These results highlight the imperative need for new studies on patient-derived proteins that may assist in redesigning structure-informed targeted therapies.

## 1. Introduction

Eradicating cancer from the body can be a clinical nightmare. The disease evolves through rounds of genetic mutations that lead healthy cells to become malignant and dangerous. As they proliferate, tumor cells become capable of colonizing and transforming their surroundings into a more suitable environment at the cost of healthy tissue. The fundamental challenge is for clinicians to destroy diseased cells that look similar to healthy cells but have ulterior motives. One solution has been to create chemotherapeutic agents with cytotoxic properties. In theory, these therapies target neoplastic growths by producing DNA damage to trigger apoptosis [1,2,3,4]. Unfortunately, cellular susceptibilities to these chemical agents vary widely from patient to patient depending on whether the apoptotic pathway has been compromised.

Years of research have uncovered important molecular culprits involved in cancer progression, with the tumor suppressor protein p53 often taking center stage. As it is implicated in ~50% of all human cancer cases, it is important to understand the structure and function of p53, and how it jeopardizes cell integrity when mutations accumulate [5,6,7]. In healthy cells p53 is primed to detect DNA damage at important cell-cycle checkpoints [8,9]. If the cell’s genetic material is deteriorated, p53 spurs apoptosis through different pathways [8,10]. One of these pathways involves the p53-mediated activation of pro-apoptotic proteins, such as PUMA [11,12]. Apoptotic markers accumulate in the mitochondria to execute the final stages of cell death [13,14]. Researchers have reported that chemotherapeutics like oxaliplatin and doxorubicin, the standard of care for advanced colorectal cancer, are ineffective against cells harboring p53 mutations [15,16,17]. Mutated p53 is less efficient at transcribing PUMA and other regulatory genes due to poor DNA promoter binding [18].

**Figure 1:**
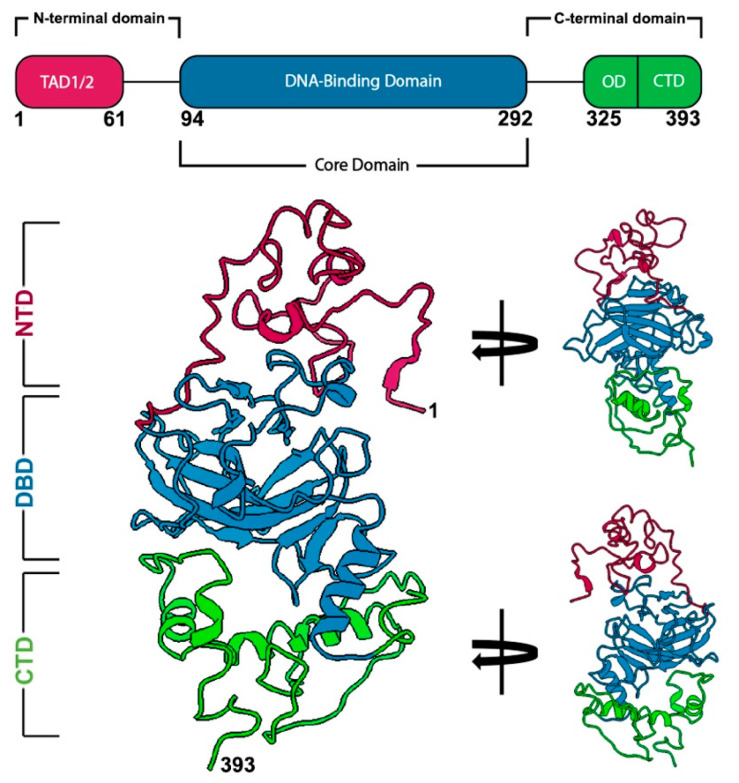
Primary sequence and structural domains of the p53 monomer. The p53 primary structure (residues 1-393) is comprised of the N-terminal domain (NTD) that contains transactivation domains (TAD 1/2), followed by the DNA-binding domain (DBD), and the C-terminal domain (CTD). Prior to the C-terminus is the oligomerization domain (OD) (top panel). The full-length p53 monomer structure was recently determined using cryo-EM and highlights each of these important regions (bottom panel, Movie S1) (pdb code, 8F2I) [22]. Length of the p53 monomer is ~60 Å.

Understanding the mechanisms by which p53 mutations impact molecular function is a high priority. As such, providing new insights for the full-length structure of p53 can elevate therapeutic design for the clinical community. Examining the primary sequence of p53, it can be divided into three main regions: a N-terminal domain (NTD) that contains transactivation domains (TAD1/2); a core domain that includes a DNA binding domain (DBD), and a C-terminal domain (CTD) that contains an oligomerization domains (OD) (Figure 1; Movie S1) [19]. Structural data for the DBD is readily available in an apo form as well as an active form bound to DNA fragments [20,21]. There remains limited information available for the flexible NTD and CTD regulatory regions, yet this information is pivotal to improve therapeutic offerings.

Recent advances in cryo-electron microscopy (EM) led us to determine the first full-length structures of p53 monomers and dimers (pdb codes 8F2I, 8F2H) [22]. Here, we expand these insights using computational approaches to reveal differences between the wild type (WT) p53 structure and models of the mutated protein. Changes in residues that effect the conformation of the DNA binding pocket were examined in comparison to the WT p53 structure. Equally important, we examined residues that may also limit protein–protein or protein-DNA interactions. By exploring the structural implications of p53 mutations, we can generate new hypotheses for biochemical testing with the potential to enhance apoptotic-based therapeutics.

## 2. Results

### 2.1. Discerning the Undiscernible: Producing p53 Models for Mutational Analysis

**Figure 2:**
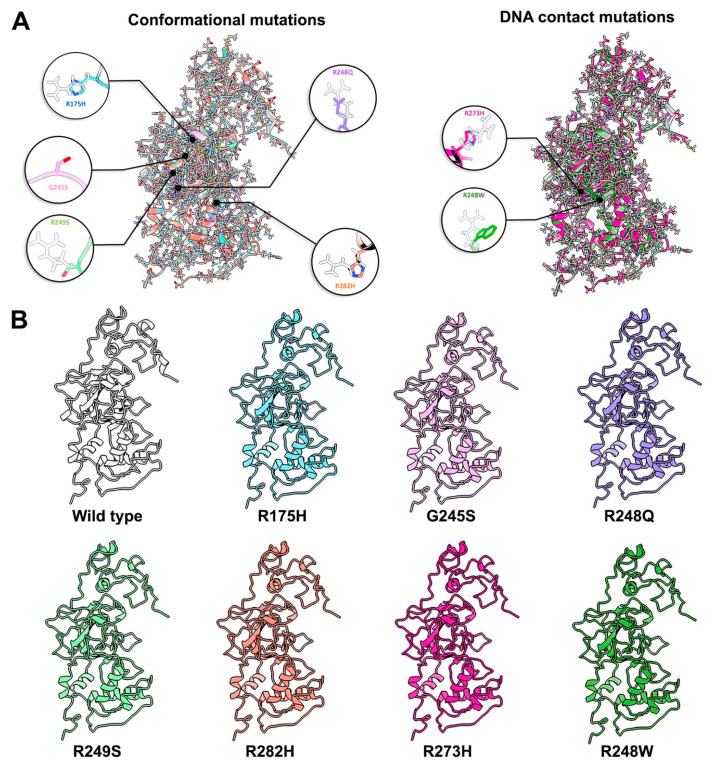
Molecular models of frequently occurring hotspot mutations in p53. (**A**) Overlay of molecular models containing hotspot mutations that affect local protein conformation or DNA contacts. Individual amino acids changes are magnified. The p53 WT model (pdb code, 8F2I) is shown in white for comparison. (**B**) Within the central DBD of p53 hotspot mutations include R175H (light blue, Movie S2), G245S (pink, Movie S3), R248Q (purple, Movie S4), R249S (light green, Movie S5), R282H (orange, Movie S6), R273H (magenta, Movie S7) and R248W (dark green, Movie S8). Percent sequence identities for each mutant was 99.75% compared with WT.

Although nearly every codon in the p53 gene contain mutations, a majority of these changes actually occur in six specific codons, referred to as “hotspots” [7,23]. These hotspot mutations are located in conserved regions of the DBD, suggesting the affected residues are important for proper functionality [5]. Some of these hotspots have been linked to poorer disease progression and chemoresistance [24,25]. Taking a closer look at the residues involved, the amino acid changes can be classified into two distinct groups: conformational mutations and DNA-contact mutations [26]. To better understand how these single point mutations in the *TP53* gene can render such grave consequences at the protein level, we implemented a molecular modeling approach in the context of the newly determined p53 structure. We employed the SWISS-MODEL server and the full-length p53 structure as a template to predict new models for seven different hotspot mutations that are most frequently found in cancer patients (Figure 2A,B; Table 1; Supplemental Movies S1–S8).

### 2.2. Conformational Mutations Have Rippling Structural Effects Due to Electrostatic Change

**Figure 3:**
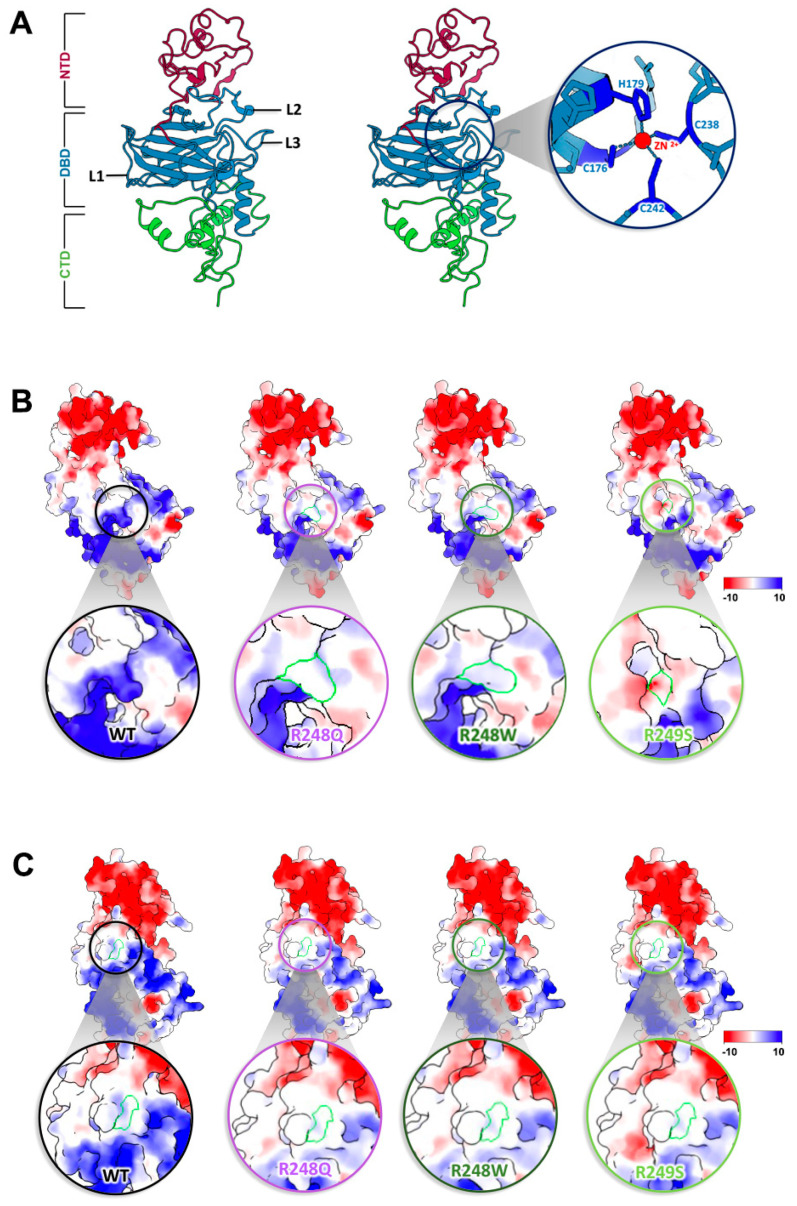
Hotspot mutations alter the electrostatic surface near a key zinc ion coordination site. (**A**) Residues in L1–L3 loop regions of the DBD provide structural integrity within the central part of the p53 protein. A magnified view of the zinc coordination site within the DBD shows key coordinating residues. (**B**) Electrostatic surface potentials mapped for residues R248Q, R248W, and R249S (highlighted with a green border) proximal to the critical zinc ion coordination site. (**C**) Surface charge potentials of an adjacent region show notable changes in point mutations with respect to WT p53. Green border indicates regions of differences. Scale for acidic to basic residues ranges from −10 (highly negative) to 10 (highly positive).

The conformational hotspot mutations we examined included residues R175H (Movie S2), G245S (Movie S3), R248Q (Movie S4), R249S (Movie S5), and R282H (Movie S6) (Figure 2) [26]. As the name suggests, these amino acid changes disrupt the local and/or global structure of p53. In broad terms, the DBD is formed by two large loops (L2 and L3) followed by a short loop-sheet-helix motif, L1 (residues 112–124) [37,38]. L2 (residues 164–194) and L3 (237–250) are regions in charge of stabilizing the zinc ion used to coordinate DNA binding (Figure 3A) [26,39,40]. Other molecular dynamics (MD) simulations predicted that the loss of the zinc ion leads a destabilization of the protein and a loss of DNA binding [41]. Most of the conformational mutations are found in the conserved regions of L3 but none of them take a part in coordinating this critical ion; instead, the residues that coordinate the zinc ion are C176, H179, C238, and C242 (Figure 3A) [42]. Our new models suggest that the loss of this zinc ion may be attributed to a change in surface charge potentials introduced by single point mutations (Figure 3B,C). By using the integrated Coulomb surface charge module in ChimeraX [43], we determined charge potentials of three hotspot mutations (R248Q, R248W, R249S) proximal to the critical ion stabilizing region of C242. The models predicted the mutations not only perturb the structural arrangement of the protein, but also introduced electrostatic changes.

In WT p53, the region is a protruding and positively charged region (Figure 3B,C). Changes in residues R248 and R249 reduced the positivity or shifted the charge to a more negative value. Cysteine has been generally classified as a polar amino acid and it creates a positive region within the zinc ion pocket. This positive pocket becomes neutral with the introduction of more neutral or negatively charged amino acids. In addition, R282 is an amino acid that is buried within the protein and is not easily accessible. The position of these amino acids shifts dramatically in comparisons to the WT structure. While biochemical experiments are needed to determine how R282 impacts protein stability, it is notable that nearby residue changes shift electrostatic charge potentials to affect zinc ion coordination.

### 2.3. Contact Mutations Are Subtle but Detrimental to Overall p53 Function

Another type of notable clinical alterations in p53 are known as DNA-contact mutations (Figure 2). These changes in DNA-binding residues disrupt p53-mediated gene expression. Two hotspots have been determined to have this ability: R273H (Movie S7) and R248W (Movie S8) [26,42]. Other studies have shown that R248W anchors the L3 loop of p53 to the minor groove of DNA, while R273H contacts the DNA backbone at the center of the p53 recognition site [26]. Modifications to these structures can have devastating consequences in patient outcomes [44].

**Figure 4:**
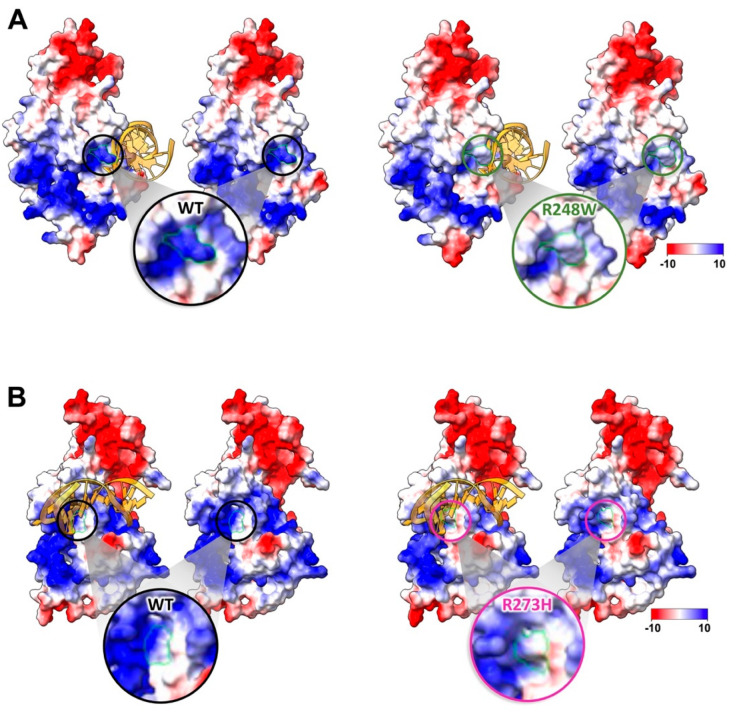
Contact mutations affect interactions with the DNA backbone through physical changes and differences in electrostatic charge potentials. Surface charge potentials are shown for residues R248W (**A**) and R273H (**B**) in the presence and absence of a DNA fragment (DNA model from pdb code, 2AC0). Magnified views show changes in hotspot residues with respect to WT p53. Green borders indicate regions of differences. Scale for acidic to basic residues ranges from −10 (highly negative) to 10 (highly positive).

Changes in R248 have mixed implications affecting both protein conformations (R248Q) and DNA contacts (R248W) [26]. For R248W, the new model revealed a major change in the DNA binding pocket of p53 (Figure 4A). In the WT structure, the arginine residue protrudes to interact with the DNA strand, as reported. The bulky tryptophan change does not have sufficient “reach” to engage the DNA strand similar to the arginine residue. Additionally, changes in the electrostatic energy profiles from positive to neutral were noted in the Coulomb surface charge analysis. This energetic change can impede the DNA backbone, which is negatively charged, from binding to p53 properly. A similar case occurs with the R273H mutation although the differences between the arginine and histidine residues are more subtle that the R248W alteration (Figure 4B). Nonetheless, the charges of the protruding residues in the R273H change also shift to more neutral in the binding pocket surface area.

**Figure 5:**
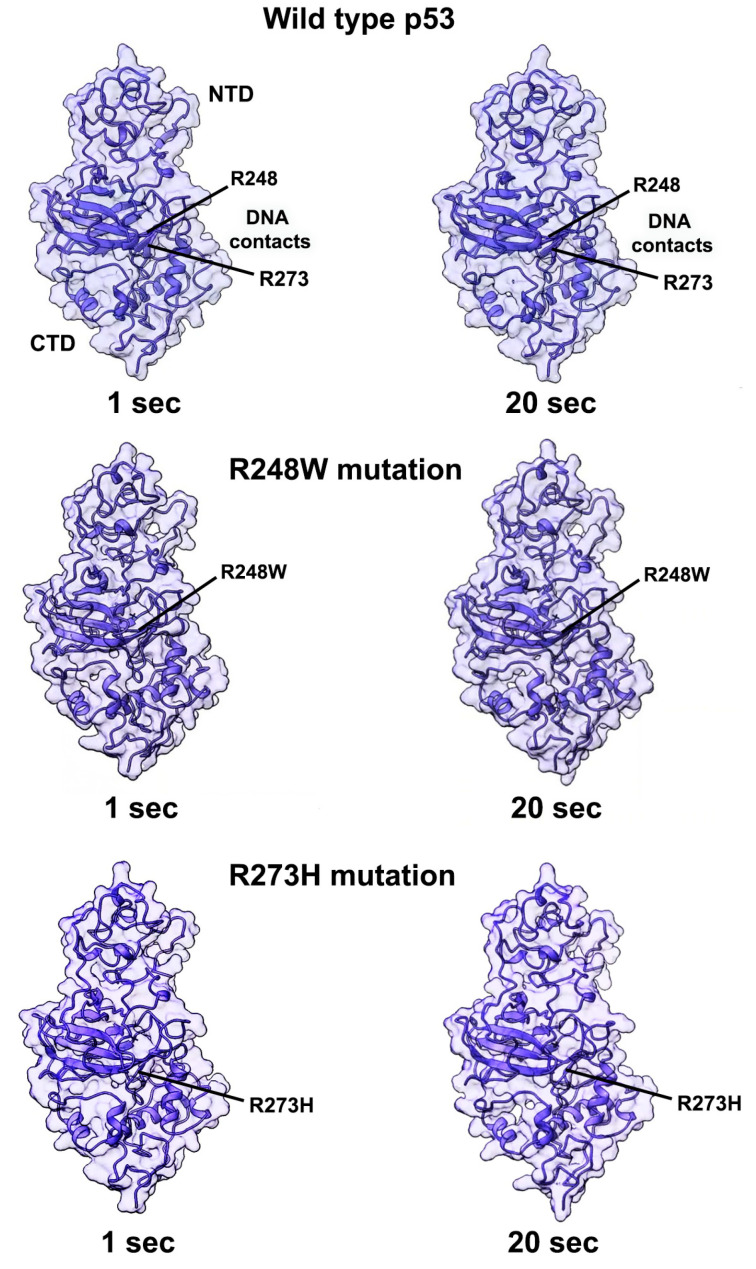
Simulations to demonstrate subtle allosteric changes in WT p53 in comparison to models of mutated p53. Dynamic simulations were performed on WT p53 along with mutated models (R248W and R273H). Movie output yielded ~20 s recordings that were accelerated 4–fold to better visualize conformational changes (Movies S9–S11). Modest changes were noted throughout each structure with less movement in the lower have of the protein (DBD and CTD), which contains more secondary structure elements. The NTD regions of each model contain more loop elements and displayed visually more motion in video recordings. Labels point to mutations in the ribbon structure.

In addition to electrostatic changes in DNA contact regions, allosteric events in other parts of the protein may affect p53’s overall performance. To gain further insight of these wholistic changes, we performed dynamic simulations of the full-length models using the flexible fitting options in the ISOLDE package integrated into ChimeraX. The simulation temperature was set at 298 K and dynamic movements of the main chain were recorded for up to 2 min for the p53 WT structure (Movie S9) along with mutated models, R248W (Movie S10) and R273H (Movie S11). Video recordings were accelerated 4-fold to better visualize changes in molecular conformations within ~20 s outputs. Continuous motion was observed throughout each p53 monomer, with less perceived motion in the lower half of the protein, even for the mutated models. This observation is likely due to the higher degree of secondary structural elements in the DBD and CTD. The NTD region, which contains extensive loop elements, appeared to exhibit greater variability. Future experimental measures of free energy and kinetic changes upon substrate binding are needed to test these computational insights and to fully quantify allosteric behaviors. The current representations of full-length p53, however, demonstrate the extent to which dynamic events can affect the DNA binding region for WT and mutated proteins alike (Figure 5; Movies S9–S11).

## 3. Discussion

The p53 protein is tightly regulated at the molecular level as it plays a pivotal role in many aspects of cellular crosstalk. Post-translational modifications (PTMs) are often employed by the cell to provide exquisite oversight of p53 during apoptosis, DNA repair, and cell cycle events. These PTMs include ubiquitination, phosphorylation, and acetylation and they are easily altered in diseased states [45,46]. The only PTM that occurs within the hotspots examined here is R249 [47,48,49]. In terms of regulatory processes, it is possible that other sites in p53 can be modified especially surface accessible residues. For instance, R248Q introduces a site that can be transformed into a lysine residue vulnerable to acetylation, phosphorylation, and ubiquitination [50,51]. Previous research has linked transglutaminase 2 to p53 by deregulating p53 expression and upregulating vascular endothelial growth factor (VEGF) to reduce apoptosis initiation [50,51]. The transformation of additional residues into serines could also affect modifications by increased potential for phosphorylated or acetylated. Currently, the PTM landscape of p53 presents a golden opportunity to modulate its function through changes in levels of corresponding protein-modifying partners.

Results from the initial modeling work presented here indicated that clinical mutations in p53 can alter surface charge potentials in key regions of the protein. A single missense mutation may affect the electrostatic climate of the whole DNA binding pocket. These changes could also prevent regulatory protein interactions and alter binding affinities in key regions, which are the subject of ongoing biochemical experiments. It is also possible that the current resolution does not lend itself to identify PTMs as EM structures present averaged ensembles of the protein population. Improved resolution and complementary experimental techniques, such as mass spectroscopy can provide a wealth of information in future investigations. These collective efforts may lead to new insights for the design of targeted drug therapies. To date, p53 has been unfortunately labelled “undruggable”. Years of effort in the pharmaceutical industry have been dedicated to designing therapeutics against p53 binding interfaces, such as MDM2 interaction sites. With the new knowledge of full-length structures in hand, these interfaces are more fully defined and can improve design efforts in light of the complete structural data.

In addition, therapeutic attempts are focusing on the unique nature of mutated p53 [52,53]. The high frequency of mutations in the *TP53* gene can decrease direct-therapy effectiveness or contribute to chemo-resistance. Functional regions may change physically or electrostatically, as previously discussed, and become less favorable binding sites for small molecule drugs. Further, each mutation can result in different loss-of-function and gain-of-function events that are only uniquely understood through a personalized assessment. These factors are further complicated by an oncological PTM landscape.

Looking ahead, it will be a valuable endeavor to resolve the full length p53 tetramer using structural techniques. P53 tetramers form exclusively on DNA strands, and a full-length model of this structure could shed light on mutational affects beyond what is predicted for the monomer structure. Full-length tetramers can also be used to better define changes in electrostatic surfaces that affect protein–protein interactions and protein-DNA events. Ultimately, improving knowledge of tumor suppressors proteins, such as p53, in any oligomeric form can provide an immense benefit for the scientific and clinical community, given that alterations in this class of proteins impacts a majority of cancer patients worldwide.

## 4. Methods and Materials

Molecular modeling and movie production. Methods to determine the p53 structure are described in previous work [22]. Maps were interpreted using a model for full-length p53 produced using the PHYRE2 Protein Fold Recognition Server [54]. Additional structures were attempted using AlphaFold but failed to produce reasonable models with known features. The initial model was first fit into the monomer map using rigid-body refinement protocols in the PHENIX software package [55]. This step required additional model rebuilding and refinement. The ISOLDE package [56] was used to perform molecular dynamics / flexible fitting on the PHENIX output to improve the quality of the model. Statistics for the final model were validated using MolProbity and previously reported [22]. The p53 model was used as template structure for additional molecular modeling experiments along with sequences for p53 point mutations. Molecular sequences and the pdb file for the WT p53 structure were submitted to the SWISS-MODEL server, outputting individual predicted structures for each missense mutation. Movies for the WT structure and each model of mutated p53 were produced using the ChimeraX software package [43]. Additional dynamic simulations were performed in ISOLDE for up to 2 min to assess structural changed in WT p53 in comparison to DNA contact hotspots (R248W, R273H). Recordings of the simulations were accelerated four-fold for visual assessment.

## Figures and Tables

**Table 1:** Clinical relevance of documented p53 hotpots mutations that affect protein conformation or DNA contacts. Mutation R175H has been found in tumors within the biliary tract, bone, brain, breast, colon/colorectum, hematopoietic system, lung, ovary, pancreas, and stomach [27,28,29,30]. Mutation G245S has been found in brain, colon/colorectum, hematopoietic system, kidney, liver, pancreas, and stomach tumors [28,29,30]. The R248Q mutation occurs in bladder, bones, brain, breast, corpus uteri, esophagus, hematopoietic system, kidney, lung, lymph nodes, pancreas, and stomach tumors [28,30,31]. The R248W mutation occurs in brain, breast, colon, esophagus, hematopoietic system, kidney, lung, lymph nodes, mouth, and skin tumors [28,30,32,33]. R249S has been documented in breast, colorectal, heart/medulla, liver, and lung tumors [28,29,30,34]. R273H has been found in the biliary tract, brain, colon/colorectum, corpus uteri, hematopoietic system, lung, lymph nodes, ovary, pancreas, skin, and thyroid tumors [27,28,29,30,35]. R282H is only documented in lung tumors [36].

Mutation	Impact on p53	Topography	Cases Referenced
R175H	Conformational	Biliary tract, bones, brain, breast, colon, hematopoietic system, lung, ovary, pancreas, stomach urinary tract	[27,28,29,30]
G245S	Conformational	Brain, colon, colorectum, liver, pancreas, hematopoietic system, stomach	[28,29,30]
R248Q	Conformational	Bladder, bones, brain, breast, corpus uteri, esophagus, hematopoietic system, kidney, lung, lymph nodes	[28,30,31]
R248W	DNA contact	Brain, breast, colon, esophagus, hematopoietic system, kidney, lung, lymph nodes, skin	[28,30,32,33]
R249S	Conformational	Breast, colorectum, heart/medulla, liver, lung	[28,29,30,34]
R273H	DNA contact	Biliary tract, brain, colon/colorectum, corpus uteri, hematopoietic system, lung, lymph nodes, ovary, pancreas, skin, thyroid	[27,28,29,30,35]
R282H	Conformational	Lung	[36]

## Data Availability

Not applicable.

## Supplementary Materials

The supporting information can be downloaded at: https://www.mdpi.com/article/10.3390/ijms232315267/s1.
